# Comparative Analysis of Umami Substances and Potential Regulatory Genes in Six Economic Bivalves

**DOI:** 10.3390/foods14244345

**Published:** 2025-12-17

**Authors:** Zheng Li, Heming Shi, Hanhan Yao, Zhihua Lin, Jiangwei Li, Yinghui Dong

**Affiliations:** 1 Key Laboratory of Aquatic Germplasm Resource of Zhejiang, College of Biological & Environmental Sciences, Zhejiang Wanli University, Ningbo 315100, China; 2 Ninghai Institute of Mariculture Breeding and Seed Industry, Zhejiang Wanli University, Ningbo 315604, China; 3 College of Advanced Agricultural Sciences, Zhejiang Wanli University, Ningbo 315101, China

**Keywords:** umami compounds, glutamate dehydrogenase 1 (*GLUD1*), bivalves, equivalent umami concentration (EUC)

## Abstract

Flavor quality fundamentally influences the market value of bivalves, yet the molecular basis of interspecific umami variation remains poorly understood, hindering flavor-directed breeding. This study compared umami compounds and related gene expression across six economically important bivalve species from Southeast China: *Crassostrea sikamea*, *Meretrix meretrix*, *M. mercenaria*, *Cyclina sinensis*, *Ruditapes philippinarum*, and *Sinonovacula constricta*. Using quantitative chemical analysis and qPCR, key taste components and gene expression levels were assessed during the peak flavor season. Results identified glutamic acid, aspartic acid, guanosine monophosphate, and adenosine monophosphate as major umami contributors. *Crassostrea sikamea* showed the highest umami intensity (Equivalent umami concentration = 449.35 g Monosodium Glutamate/100 g dry weight), followed by *Meretrix meretrix* (EUC = 329.56 g MSG/100 g dry weight). Expression of glutamate dehydrogenase 1 strongly correlated with glutamic acid content (r = 0.90, *p* < 0.01), indicating its regulatory role. glutamic-oxaloacetic transaminase 1 and adenylosuccinate synthase also associated positively with aspartic and glutamic acids, respectively, while hypoxanthine phosphoribosyltransferase 1 correlated negatively. Bioinformatics revealed species-specific variations in key enzyme active sites. This study integrates flavor phenotyping with genetic analysis, offering novel insights into umami regulation and providing candidate genes for molecular breeding aimed at flavor enhancement, but subject to further functional validation and heritability analysis.

## 1. Introduction

Marine bivalves have become an important component of global aquaculture and seafood consumption due to their unique oceanic umami taste and high nutritional [1,2,3]. In China, farmed bivalves account for approximately 69.85% of the total mariculture production. Their distinct flavor profile, particularly the richness and pleasantness of umami, is a key quality attribute determining consumer acceptance and market [4].

As one of the basic tastes, umami is primarily mediated by free amino acids (such as glutamate and aspartate) and flavor nucleotides [including 5′-guanosine monophosphate (GMP) and 5′-adenosine monophosphate (AMP)]. These compounds not only directly contribute to taste but also synergistically enhance overall umami perception [5,6]. In recent years, against the backdrop of growing consumer demand and the development of flavor-oriented breeding strategies, elucidating the material basis and molecular mechanisms underlying umami formation in bivalves has emerged as a frontier research topic at the intersection of food science and aquaculture.

Researchers have made significant progress in characterizing the umami profiles of bivalves, employing techniques such as high-performance liquid chromatography (HPLC), amino acid analysis, and electronic tongue systems to quantify taste-active compounds across different species, rearing environments, and processing methods [7,8,9]. The equivalent umami concentration (EUC) has been widely adopted as a reliable indicator for assessing umami intensity [5,9,10]. These studies consistently report significant inter- and intraspecies variations in the concentrations of umami compounds, with oysters and hard clams often exhibiting higher EUC values [11,12]. Furthermore, glutamate content varies markedly among populations, and AMP levels show distinct seasonal fluctuations [13,14,15].

Although previous studies have clearly documented differences in umami compound composition and their correlation with perceived umami intensity, the molecular and genetic mechanisms controlling the biosynthesis, accumulation, and regulation of these key tastants remain largely unknown. This knowledge gap represents a major obstacle to genetically improving flavor traits in bivalves through molecular breeding [16]. The metabolism of umami compounds involves a complex biochemical network. The synthesis and interconversion of glutamate and aspartate are closely linked to the activities of transaminases (e.g., *GOT1*) and dehydrogenases (e.g., *GLUD1*) [17]. The intracellular levels of flavor related nucleotides such as GMP and AMP reflect the balance between de novo and salvage synthesis, which is tightly controlled by key enzymes including adenylosuccinate synthase (*ADSS*) and hypoxanthine phosphoribo-syltransferase 1 (*HPRT1*) [18]. In teleost fish, the roles of these genes in muscle flavor have begun to be clarified. Glutamate elevates IMP in triploid crucian carp by up-regulating PPAT-mediated de novo synthesis and increasing ATP supply, and specific SNPs in AMPD1 are associated with higher IMP content in hybrid sea bream [19,20]. Whether these same loci govern umami traits in bivalves and other invertebrates is, however, still unknown. Pinpointing the genes that act as candidate regulators and defining how their expression relates to taste compound accumulation are essential steps toward unraveling the molecular basis of flavor formation in bivalves.

Against this background, we hypothesized that the interspecies differences in umami intensity among economic bivalves arise not only from variations in the content of taste compounds but are also closely related to the expression levels of key genes regulating their synthesis and metabolism. To test this hypothesis, we selected four economically important bivalve species farmed in Zhejiang Province, China—the Asian clam (*Cyclina sinensis*), hard clam (*Meretrix meretrix*), constricted tagelus (*Sinonovacula constricta*), Asian green clam (*C. sinensis*), Manila clam (*Ruditapes philippinarum*), and razor clam (*S. constricta*)—as research models. Using an integrated approach that combined taste compound quantification with EUC calculation, gene family identification and structural analysis, and qPCR-based expression profiling, we first systematically compared the composition and taste activity of umami-related amino acids and nucleotides across these species. We then proceeded to identify and characterize the sequence features and cross-species conservation of four key candidate genes (*GLUD1*, *GOT1*, *ADSS*, *HPRT1*). Finally, we analyzed the correlation between the expression levels of these genes and the content of major umami substances. Our findings are expected to provide initial insights into the molecular regulatory basis of umami compound accumulation in bivalves, thereby establishing a theoretical foundation and identifying genetic targets for molecular marker-assisted breeding aimed at improving flavor quality.

## 2. Materials and Methods

### 2.1. Materials and Regents

To ensure high levels of umami-related compounds, all samples were collected during the documented seasonal peak for free amino acids such as glycine and glutamate [20,21,22,23,24,25]. The six species of shellfish were all from Lulin Aquatic Market in Ningbo, Zhejiang Province, China (Table 1).

For each species, ten healthy individuals of comparable size (see Table A1 for average specifications) were randomly selected. These individuals were first acclimated under specific temporary conditions: a temperature of 20 °C and salinities tailored to each species (*M. meretrix*: 24‰ (parts per thousand); *C. sinensis*: 22‰; *R. philippinarum*: 24‰; *M. mercenaria*: 26‰; *C. sikamea*: 26‰; *S. constricta*: 20‰). Following this, they underwent a 24 h depuration process in filtered seawater to clear sediment and metabolic waste. Subsequently, the shells were manually removed, and the muscle tissues were dissected. The collected tissues were immediately flash-frozen in liquid nitrogen and stored at −80 °C until further analysis. This sampling scheme established ten biological replicates for each species.

Key apparatus employed in this study included the L-8800 Amino Acid Analyzer (Hitachi High-Tech Corporation, Tokyo, Japan), Agilent 1260 HPLC system (Agilent Technologies, Santa Clara, CA, USA), CFX96 Real-Time PCR System (Bio-Rad Laboratories, Hercules, CA, USA), JXFSTPRP-64L Automated Sample Rapid Grinder (Shanghai Jingxin Industrial Development Co., Ltd., Shanghai, China), FreeZone 4.5 L Freeze Dryer (Labconco Corporation, Kansas City, MO, USA), and M1324R Refrigerated Microcentrifuge (RWD Life Science Co., Ltd., Shenzhen, China).

### 2.2. Measurement of Umami Substances

#### 2.2.1. Determination of Free Amino Acids Content

Following vacuum freeze-drying, the edible tissues were ground into a fine powder using a homogenizer. A 0.1 g portion of the muscle tissue powder was precisely weighed and homogenized with 5 mL of 0.01 M HCl containing 0.2% (*v*/*v*) trichloroacetic acid (TCA), achieving a solid-to-liquid ratio of 1:50 (g/mL). The mixture was then subjected to ultrasonic extraction for 10–15 min in an ice bath. After ultrasonication, the extract was mixed with an equal volume of 8% sulfosalicylic acid solution and incubated at 4 °C for 30 min to precipitate proteins. The sample was subsequently centrifuged at 3000× *g* and 4 °C for 30 min. The supernatant was collected, passed through a 0.22 μm membrane filter, and the filtrate was transferred into a 2 mL injection vial for analysis. Amino acid separation was performed using an ion-exchange resin column (4.6 mm × 60 mm) maintained at 57 °C. A 20 μL aliquot of the prepared sample was injected and eluted with a lithium salt buffer (pH 2.8–4.2) at a constant flow rate of 0.40 mL/min. Post-column derivatization was carried out with ninhydrin in a reaction chamber heated to 130 °C, and the derivatives were detected at a wavelength of 570 nm.

#### 2.2.2. Determination of Free Nucleotides

A 0.1 g aliquot of the edible part powder was accurately weighed and mixed with 1 mL of 10% perchloric acid solution. The mixture was vortexed for 1 min and then subjected to ultrasonic extraction for 10 min in an ice bath. After centrifugation at 8000× *g* and 4 °C for 10 min, the supernatant was collected. The resulting precipitate was re-extracted with another 1 mL of 10% perchloric acid, and the supernatants were combined. The pH of the pooled supernatant was carefully adjusted to 6.0–6.4 under ice-bath cooling, using sequential additions of 10 mol/L and 1 mol/L NaOH, followed by centrifugation at 12,000× *g* and 4 °C for 15 min. The resulting supernatant was passed through a 0.22 μm membrane filter prior to HPLC analysis. Separation was performed on a C18 column with a mobile phase consisting of a gradient of methanol and 10 mM KH_2_PO_4_ containing 4 mM tetrabutylammonium hydrogen sulfate (TBHS, pH 6.0). Detection was carried out at 254 nm. Calibration curves were established using standard solutions of 5′-AMP, 5′-IMP, and 5′-GMP in the concentration range of 0.1–50 μg/mL (all with R^2^ > 0.995). The content of each nucleotide was calculated according to formula:

Nucleotide content (μg/g) = C × V/W(1)

where “C” is HPLC determination of concentration, μg/mL; “V” is extract volume, mL; “W” is Sample mass.

#### 2.2.3. Taste Intensity Value and MSG Equivalent

The taste activity value (TAV) was employed to evaluate the contribution of individual taste compounds to the overall flavor profile of the six bivalve species. The TAV was calculated using Equation (2) as the ratio of the concentration of a compound to its recognized taste threshold. The gustatory thresholds for amino acids were adopted from the literature [26] as follows: umami amino acids—glutamate (Glu, 30 mg/100 g) and aspartate (Asp, 10 mg/100 g); sweet amino acids—alanine (Ala, 6 mg/100 g), glycine (Gly, 13 mg/100 g), threonine (Thr, 26 mg/100 g), and serine (Ser, 15 mg/100 g); bitter amino acids—arginine (Arg, 50 mg/100 g), histidine (His, 20 mg/100 g), and valine (Val, 15 mg/100 g); and unflavored amino acids—lysine (Lys, 5 mg/100 g) and proline (Pro, 30 mg/100 g). For the nucleotides 5′-GMP and 5′-AMP, the taste thresholds of 12.5 mg/100 g and 50 mg/100 g, respectively, were referenced from Yamaguchi et al. [15,20].

TAV = C/T(2)

where “C” represents the absolute concentration value of taste substances, and “T” represents the threshold value of taste substances.

Based on the measured contents of umami nucleotides (IMP + GMP) and free amino acids (Glu + Asp), the equivalent umami concentration (EUC) was determined according to Equation (3) and expressed as grams of monosodium glutamate per 100 g of sample (g MSG/100 g dry weight).

EUC (g MSG/100 g dry weight) = ∑ aibi + 1218 (∑ aibi) (∑ ajbj)(3)

where “ai” represents the concentration (g/100 g) of an umami amino acid (aspartic acid or glutamic acid); “bi” denotes its relative umami potency compared to monosodium glutamate (MSG), with values of 1.000 for glutamic acid and 0.077 for aspartic acid; “aj” is the concentration (g/100 g) of a flavor nucleotide (5′-AMP, 5′-IMP, or 5′-GMP); and “bj” is the relative umami potency of the nucleotide compared to MSG, with values of 0.18 for 5′-AMP, 1.0 for 5′-IMP, and 2.3 for 5′-GMP. The constant “1218” represents the synergistic coefficient [5].

### 2.3. Identification and Validation of Candidate Genes

#### 2.3.1. Candidate Gene Selection and Bioinformatic Analysis

The coding sequences of the target genes (*GOT1*, *GLUD1*, *ADSS*, and *HPRT1*) for *R. philippinarum*, *C. sinensis*, and *M. mercenaria* were retrieved from the NCBI RefSeq database, while those for *M. meretrix*, *S. constricta*, and *C. sikamea* were derived from in-house genome/transcriptome assemblies. Multiple amino acid sequence alignment was performed using the ClustalW module in MEGA 11 with a gap opening penalty of 10, and the resulting alignment was edited using GeneDOC (version 2.7). Tertiary protein structures were predicted via the AlphaFold Server and visualized in PyMOL v3.1. A maximum likelihood phylogenetic tree was constructed under the LG + G4 model, with branch support evaluated using 1000 bootstrap replicates. Conserved motifs were identified with the MEME online tool, and gene structures were visualized in TBtools v1.132 [27] based on species-specific GFF annotation files.

#### 2.3.2. Determination of Umami-Related Gene Expression

Total RNA was extracted from the edible part using the TRIzol reagent. cDNA was then synthesized from the extracted RNA following the instructions of the HiScript II Q RT SuperMix for qPCR (+gDNA wiper) kit (Novozymes, Nanjing, Jiangsu Province, China). Quantitative PCR was performed using the ChamQ SYBR qPCR Master Mix (Novozan, Nanjing, Jiangsu Province, China) according to the manufacturer’s protocol. The following genes were used as internal controls: *R. philippinarum* (*RpL3*), *C. sinensis* (*β-actin-1*), *M. meretrix* (*β-actin-2*), *C. sikamea* (*EF1α-1*), *S. constricta* (*RS9*), and *M. mercenaria* (*EF1α-2*) (Table 2). A no-template control (DNA-free reaction) was included in each run. Prior to expression analysis, primer specificity and amplification efficiency were validated by constructing a standard curve using serial dilutions of cDNA.

### 2.4. Statistical Analysis

All data are expressed as the mean ± standard deviation from at least three independent replicates. Statistical significance was assessed by one-way ANOVA followed by post hoc tests using SPSS 24.0, with a *p*-value < 0.05 considered statistically significant. The relative mRNA expression levels of target genes were determined by quantitative PCR and calculated via the 2^−ΔΔCT^ method. Pearson correlation analysis was employed to examine potential associations between gene expression levels and the content or taste activity value (TAV) of umami-related compounds. All figures and correlation plots were generated using GraphPad Prism 8, PyMOL 3.1, and RStudio (version 2024.12.0+467 ‘Kousa Dogwood’, Posit Software, PBC).

## 3. Results

### 3.1. Flavor Characteristics and Taste Contribution of Amino Acids

#### 3.1.1. Free Amino Acid

**Figure 1:**
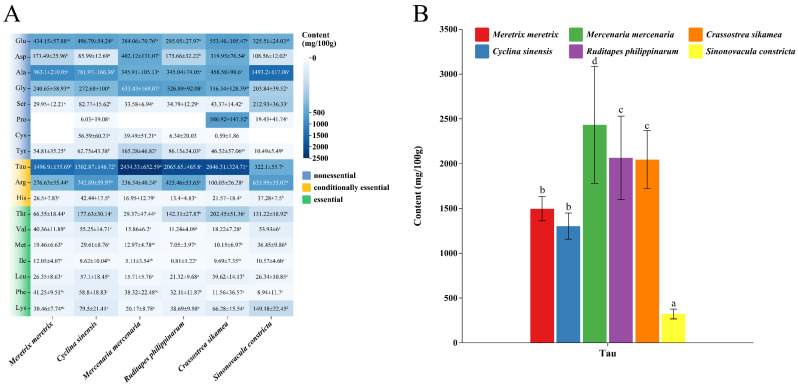
Free amino acid and taurine profiles in six bivalve species. (**A**) Heatmap visualization of free amino acid concentrations. The color intensity from blue to white represents the concentration gradient. (**B**) Taurine concentrations across species. Bars sharing different letters differ significantly based on Tukey’s HSD test (*p* < 0.05). Error bars represent mean ± SD (*n* ≥ 3).

As shown in Table 3, the total free amino acid (TFAA) content exhibited significant interspecific variation (*p* < 0.05). Numerically, *C. sikamea* (4851.66 mg/100 g) and *M. mercenaria* (4815.79 mg/100 g) ranked the highest, followed by *C. sinensis* (4400.31 mg/100 g) and *R. philippinarum* (4217.96 mg/100 g), while *S. constricta* recorded the lowest content (3783.93 mg/100 g). Analysis of the specific amino acid profiles (Figure 1A) identified six predominant compounds across all species: glutamate, aspartate, alanine, glycine, taurine, and arginine. Notably, cysteine was detected only sporadically in trace amounts. Proline showed distinctive distribution patterns, being abundant in *C. sikamea* but virtually undetectable in *C. sinensis* and *S. constricta*. Regarding taurine, remarkable interspecific differences were observed (Figure 1B). *M. mercenaria* exhibited the highest concentration (2434.33 mg/100 g), which was significantly higher (*p* < 0.05) than that of the intermediate group formed by *C. sikamea* and *R. philippinarum*. In contrast, *S. constricta* contained the lowest taurine level (322.10 mg/100 g), significantly differing from all other species.

#### 3.1.2. Taste Evaluation of Amino Acids

**Figure 2:**
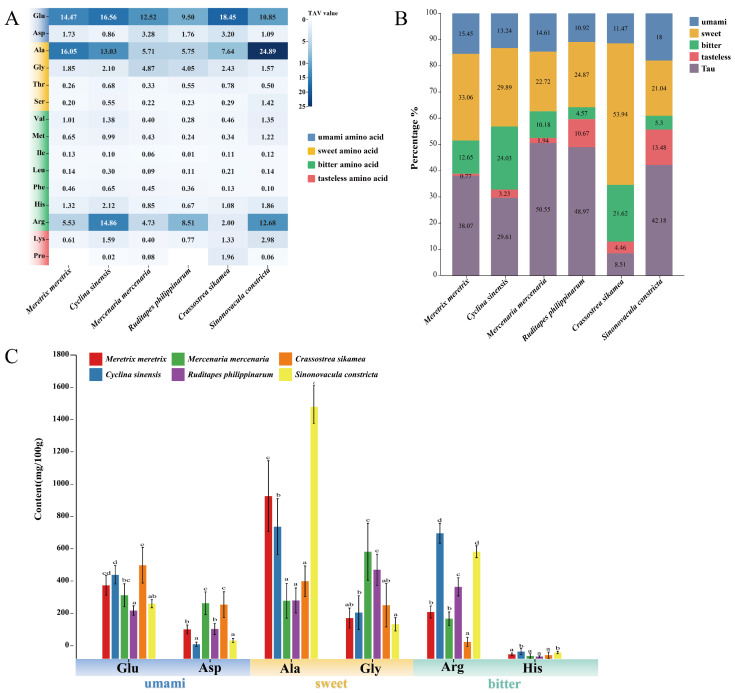
Taste activity and composition of key flavor-related amino acids in six bivalve species. (**A**) Heatmap of Taste Activity Values (TAVs). TAV was calculated as the ratio of amino acid concentration to its taste threshold [5]. Color intensity reflects TAV magnitude; TAV ≥ 1 indicates a meaningful contribution to taste. (**B**) Proportional composition of major flavor-related amino acids, including taurine (Tau), across species. (**C**) Statistical comparison of content levels for individual flavor amino acids. Bars sharing the same letter are not significantly different (Tukey’s HSD test, *p* < 0.05). Error bars represent mean ± SD (*n* ≥ 3).

As shown in Figure 2A, glutamate (Glu) exhibited the highest taste activity value (TAV) among umami amino acids in *C. sikamea* (18.45). Among the four clam species, Glu TAV levels followed a descending order: *C. sinensis* (16.56), followed by *M. meretrix* (14.47), *M. mercenaria* (12.52), and *R. philippinarum* (9.50). Aspartate (Asp) TAV was highest in *M. mercenaria* (3.28) and *C. sikamea* (3.20), while *C. sinensis* exhibited the lowest value (0.86). The mean TAV of Glu across all six species was 14.23, indicating its dominant role in direct umami taste, while Asp, with a mean TAV of 1.99, served as a supplementary contributor.

Among sweet amino acids, alanine (Ala) showed the highest TAV in *S. constricta* (24.89), followed by *M. meretrix* (16.05), *C. sinensis* (13.03), and *C. sikamea* (7.64). The lowest Ala TAVs were observed in *R. philippinarum* (5.75) and *M. mercenaria* (5.71). Glycine (Gly) TAVs also exceeded 1 in all species, suggesting a consistent contribution to sweet taste. The proportional composition of flavor amino acids relative to total free amino acids varied across species (Figure 2B). *C. sikamea* had the highest proportion of umami amino acids, whereas *S. constricta* showed the highest proportion of sweet amino acids. Among the four clam species, *M. meretrix* contained the highest combined proportion of umami and sweet amino acids. As illustrated in Figure 2C, arginine (Arg) was the third most abundant flavor amino acid after Glu and Ala, with a mean TAV of 8.05 across species. Statistically, Arg levels were highest in *C. sinensis* (14.86) and *S. constricta* (12.68) (*p* < 0.05), followed by *R. philippinarum* (8.51), which formed a distinct intermediate tier. *M. meretrix* (5.53) and *M. mercenaria* (4.73) showed significantly lower levels, while *C. sikamea* (2.00) was the lowest (*p* < 0.05). This variation suggests Arg plays a potential umami-enhancing role. The remaining flavor amino acids generally showed TAVs between 0.1 and 1, implying a minor role in direct taste but a possible modulating influence on overall flavor.

### 3.2. Umami Intensity and Synergistic Effect of Nucleotides

#### 3.2.1. Flavor Nucleotide

**Figure 3:**
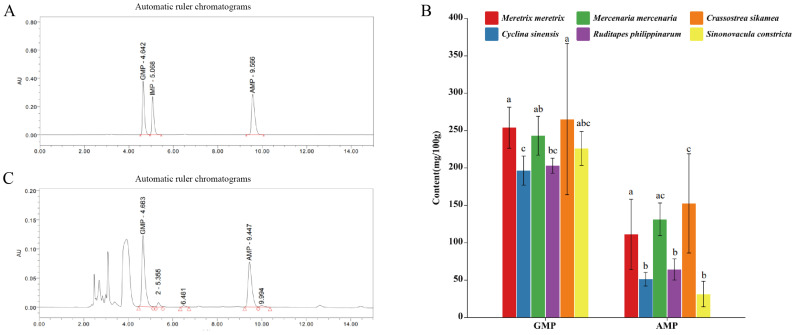
Chromatograms and nucleotide contents of six bivalve species. (**A**) Representative HPLC chromatograms of standards and samples. (**B**) Contents of GMP and AMP in six bivalve species. (**C**) Enlarged view of a representative HPLC chromatogram, illustrating nucleotide peaks in a bivalve sample. Letter markers indicate significant differences based on Tukey’s HSD test (*p* < 0.05); bars sharing the same letter are not statistically different. Error bars represent mean ± SD (*n* ≥ 3).

The synergistic interactions among umami compounds can substantially enhance overall umami perception. As summarized in Table 4, both GMP and AMP were detected in all six bivalve species, whereas IMP was not identified in any sample. Among the flavor nucleotides, GMP was present at the highest concentration and represented the predominant nucleotide contributor to taste. As shown in Figure 3B, although *C. sikamea* exhibited the numerically highest GMP content (265.17 mg/100 g), it was not statistically different from that of *M. meretrix* (253.83 mg/100 g, *p* > 0.05). Overall, GMP levels exhibited a decreasing trend from *C. sikamea* to *C. sinensis*, with *C. sinensis* presenting significantly lower levels (196.48 mg/100 g) compared to the top-ranking species. Regarding AMP, *C. sikamea* also displayed the highest concentration (152.49 mg/100 g), which was statistically comparable to *M. mercenaria* (131.29 mg/100 g) but significantly higher than the other four species (*p* < 0.05). Conversely, *C. sinensis*, *R. philippinarum*, and *S. constricta* contained the lowest AMP levels (<65 mg/100 g). A comparative analysis of the four clam species revealed that while *M. meretrix* and *M. mercenaria* generally contained higher nucleotide levels than *C. sinensis* and *R. philippinarum*, statistically significant differences were more pronounced in AMP than in GMP. Consistent with the TAV results, GMP was consistently more abundant than AMP across all species.

#### 3.2.2. Nucleotide Taste Evaluation and Monosodium Glutamate Equivalent

As shown in Table 5, the taste activity values (TAVs) of the umami nucleotide GMP varied significantly among species. *C. sikamea* (21.21) and *M. meretrix* (20.31) exhibited the highest values, followed by *M. mercenaria* (19.45) and *S. constricta* (18.09). The mean GMP TAV across all six bivalve species was 18.50, indicating that GMP serves as the primary nucleotide contributing directly to umami taste. Regarding AMP, TAVs exceeded 1 in five of the six species, with the exception of *S. constricta* (0.63), suggesting a potential auxiliary role in umami enhancement. Unlike GMP, distinct interspecific differences were observed in AMP TAVs, with *C. sikamea* showing significantly higher levels compared to *C. sinensis* and *R. philippinarum*. Among the four clam species examined, *M. meretrix* exhibited the highest numerical GMP TAV (20.31), although it was not statistically different from that of *M. mercenaria*.

The equivalent umami concentration (EUC) quantifies the umami intensity resulting from the synergistic interaction between umami amino acids and flavor nucleotides, expressed as the concentration of monosodium glutamate (MSG) producing an equivalent taste response. This synergy, primarily driven by glutamate, aspartate, AMP, and GMP, plays a central role in enhancing the umami perception of bivalves. As summarized in Table 6, the EUC values varied considerably among the six bivalve species, in the following descending order: *C. sikamea* (449.35 g/100 g), *M. meretrix* (329.56 g/100 g), *M. mercenaria* (284.93 g/100 g), *C. sinensis* (283.23 g/100 g), *S. constricta* (214.08 g/100 g), and *R. philippinarum* (174.32 g/100 g). Statistical analysis indicated that the EUC of *C. sikamea* was higher than those of the other five species. Among the four clam species, *M. meretrix* exhibited a greater EUC than the other three clams. The EUC results demonstrate that *C. sikamea* possesses the most intense umami flavor among the six economically important bivalves studied, whereas *M. meretrix* ranks highest in umami intensity within the clam group.

### 3.3. Evolutionary, Expression and Correlation Analyses of Umami Gene

#### 3.3.1. Multiple Sequence Alignment Analysis

**Figure 4:**
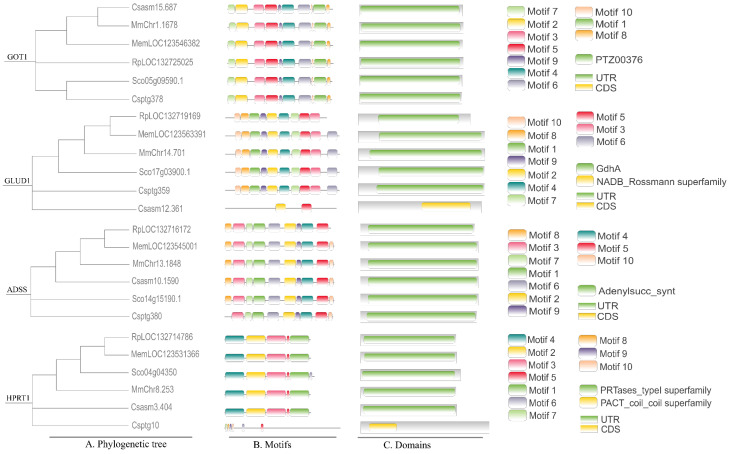
Comparative genomic analysis of six bivalves. Rp: *R. philippinarum*, Cs: *C. sinensis*, Mm: *M. meretrix*, Cp: *C. sikamea*, Sc: *S. constricta*, Me: *M. mercenaria*.

Based on integrated KEGG pathway enrichment and genomic database analysis, we identified four key rate-limiting enzymes involved in the synthesis and metabolism of umami compounds: glutamate-oxaloacetate transaminase (GOT), glutamate dehydrogenase (GLUD), adenylosuccinate synthase (ADSS), and hypoxanthine phosphoribosyltransferase (HPRT) (Figure 4). Bioinformatics analysis revealed conserved domain architectures across the six bivalve species. The *GOT1* gene consistently encoded a protein belonging to the PTZ00376 superfamily. In contrast, the *GLUD1* gene encoded a NADB_Rossmann superfamily protein in *C. sinensis*, whereas it was classified under the GdhA superfamily in the other five species. *ADSS* genes were highly conserved, all falling within the adenylosucc_synt superfamily. *HPRT1* was assigned to the PRTases_type1 superfamily in most species, with the exception of *C. sikamea*, where it was identified as a member of the PACT_coil superfamily.

**Figure 5:**
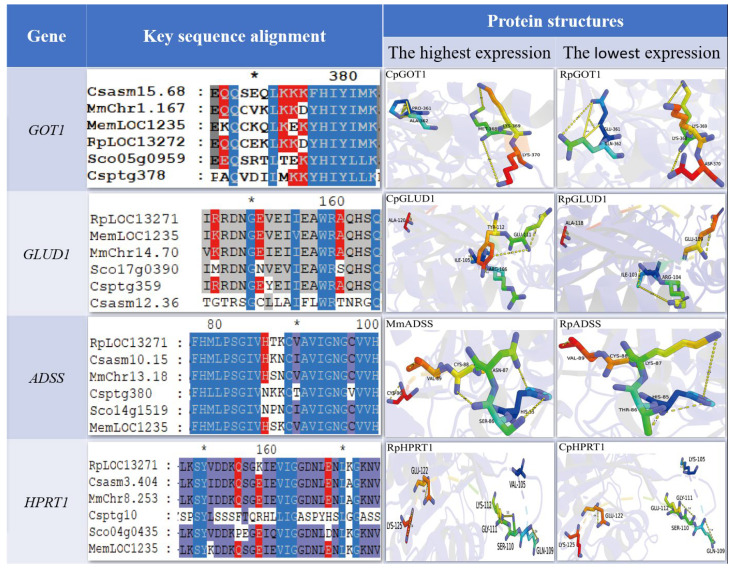
Comparative analysis of positively selected residues and tertiary architectures of four metabolism-critical genes (*GOT1*, *GLUD1*, *ADSS* and *HPRT1*) across six commercially important bivalve species. Rp: *R. philippinarum*, Cs: *C. sinensis*, Mm: *M. meretrix*, Cp: *C. sikamea*, Sc: *S. constricta*, Me: *M. mercenaria*. Color structure represents the key residues of the active site (stick model), yellow dotted line represents hydrogen bond, and purple band represents the overall structure of the target protein.

In addition, we predicted the tertiary structure of these four genes that may be related to umami. Sequence alignment and tertiary structure model showed that *GOT1* gene formed catalytic active sites composed of two Gln (Q) and three Lys (K) in *C. sinensis* in six economic shellfishes (Figure 5). Conservative Lys (K) → Asp (D) substitution occurred between *M. meretrix* and *R. philippinarum*. *C. sikamea* had Gln (Q) → Ala (A) and Lys (K) → Met (M) double mutations. *S. constricta* had Lys (K) → Thr (T)/Glu (E) substitution. Its rod-like domain is highly conserved in *R. philippinarum*, while *C. sikamea* exhibits a unique ring conformation. The *GLUD1* gene in *R. philippinarum* maintained a conserved catalytic residue and rod-like structure; *C. sikamea* contains ring-like structural features. The residue conformation of *ADSS* gene in two clams (*M. meratrix* and *R. philippinarum*) was highly similar, while *C. sikamea* had His (H) → Asn (N) and Cys (C) → Val (V) substitutions, and *S. constricta* had only His (H) → Asn (N) single point mutation. The *HPRT1* gene showed different evolutionary patterns: *C. sikamea* had significant variations in catalytic residues and tertiary structure, and *R. philippinarum* maintained a high degree of homology. The structural variation of these genes may be one of the factors that cause the differences in the characteristic umami substances of the six shellfishes.

#### 3.3.2. Analysis of Umami-Related Gene Expression

**Figure 6:**
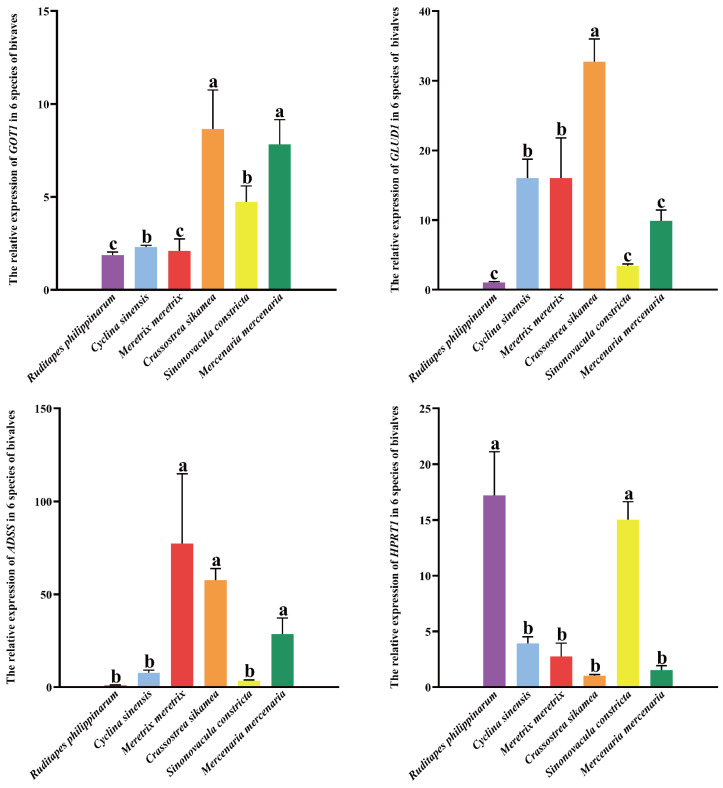
The relative expression of four genes in six species of bivalves. The values in the cells were mean ± SD (*n* ≥ 3), and the letters indicated Tukey HSD grouping (there was no significant difference between the same letters, *p* > 0.05).

Quantitative PCR analysis was performed to determine the expression levels of four key genes (*GOT1*, *GLUD1*, *ADSS*, and *HPRT1*) in the six bivalve species. As illustrated in Figure 6, *GLUD1* expression was significantly highest in *C. sikamea* (*p* < 0.01), followed by *M. meretrix*, *C. sinensis*, *M. mercenaria*, and *S. constricta*, with the lowest level observed in *R. philippinarum*. *C. sikamea* showed significantly higher *GLUD1* expression than the other five species (*p* < 0.05). Similarly, *GOT1* expression was highest in *C. sikamea* (*p* < 0.05), followed by *M. mercenaria*, *S. constricta*, *C. sinensis*, and *M. meretrix*, and again lowest in *R. philippinarum*. No significant difference was detected between *C. sikamea* and *M. mercenaria*, whereas *C. sikamea* differed significantly from the remaining four species.

#### 3.3.3. Pearson Correlation Analysis

**Figure 7:**
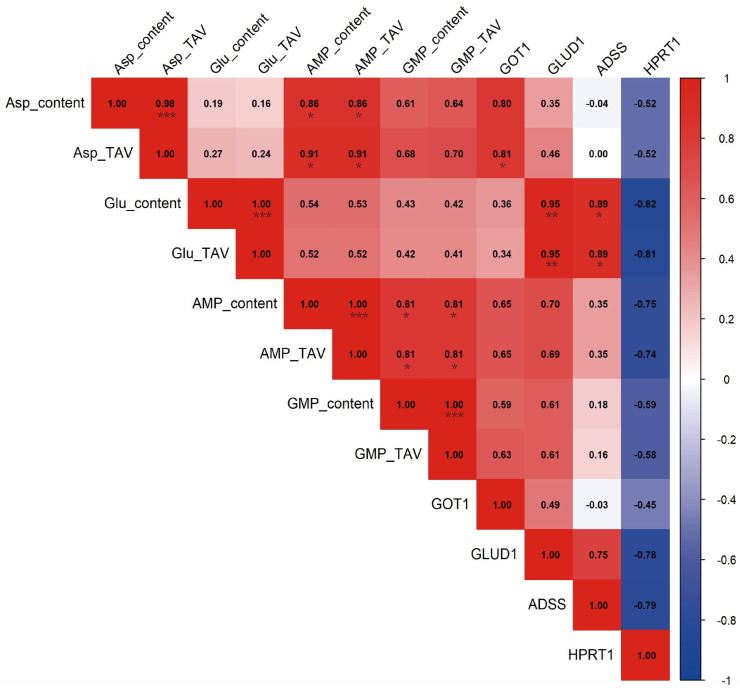
The correlation between umami substances and gene expression was calculated using Pearson’s correlation coefficient. The square number represents the correlation value, * represents the significant difference, * *p* < 0.05, ** *p* < 0.01, *** *p* < 0.001. The background color from shallow to deep indicates that the correlation from small to large, red represents a positive correlation, and blue represents a negative correlation.

Pearson correlation analysis was performed to assess relationships between the expression levels of key genes and the contents of umami-related compounds in the six bivalve species (Figure 7). *GLUD1* expression showed a significant positive correlation with both glutamate content and its taste activity value (TAV) (*p* < 0.05). *GOT1* expression was significantly correlated with the TAV of aspartate (*p* < 0.05), indicating a potential role in regulating aspartate-derived umami, whereas no significant correlation was observed with glutamate content, glutamate TAV, or nucleotide levels. *ADSS* expression was significantly positively correlated with glutamate content and TAV (*p* < 0.05), and exhibited only weak correlations with other umami compounds, suggesting functional specificity toward glutamate metabolism. In contrast, *HPRT1* expression was significantly negatively correlated with glutamate content and TAV (*p* < 0.05), and showed generally weaker negative associations with other umami indicators.

## 4. Discussion

### 4.1. Analysis of Amino Acid Content Characteristics of Six Bivalves

Umami is widely recognized as one of the five basic taste modalities, alongside sweet, sour, salty, and bitter [28,29,30,31]. In marine organisms, the umami characteristic is primarily derived from free amino acids (FAAs) and 5′-ribonucleotides, with L-glutamic acid (Glu) and aspartic acid (Asp) acting as the predominant agonists of the T1R1/T1R3 heterodimer [28,32]. In the present study, the equivalent umami concentration (EUC) varied significantly among the six bivalve species, ranging from 214.08 to 449.35 g MSG/100 g dry weight. Notably, inter-specific differences in FAA composition accounted for 87% of this variation (PERMANOVA, *p* < 0.001), with glutamic acid identified as the single most influential determinant. *C. sikamea* contained 553.46 mg Glu 100 g^−1^, yielding a taste activity value (TAV) of 18.45, which exceeded the human sensory threshold by approximately 18-fold. Crucially, Glu abundance was strongly and positively correlated with the transcript levels of *GLUD1* (r = 0.952, *p* < 0.01), the mitochondrial enzyme responsible for catalyzing the final oxidative deamination of Glu. This transcription–metabolite covariance provides compelling evidence that *GLUD1* expression serves as a rate-limiting node governing umami intensity, thereby explaining why *C. sikamea* exhibited the highest EUC (449.35 g MSG/100 g dry weight). Conversely, *S. constricta* and *R. philippinarum*, which contained 325.51 and 285.03 mg Glu 100 g^−1^, respectively, displayed proportionally lower EUC values (294.8 and 267.4 g MSG/100 g dry weight), a finding that corroborates previous observations in *C. gigas*, *M. meretrix*, and *C. farreri* [8,9].

Although present at lower concentrations than Glu, Asp also contributed appreciably to umami, with TAVs > 1 in all species except *C. sinensis* (TAV = 0.87). The synergistic interplay between Asp and Glu serves to amplify overall palatability via receptor-mediated signaling [33]. In addition to these primary umami agents, variations in sweet and bitter FAAs modulate flavor perception, creating distinctive taste profiles for each species [34,35]. Alanine (Ala), which imparts a pleasant sweet note, dominated the FAA profile of *M. meretrix* and *S. constricta* (TAV = 16.05 and 24.89, respectively), conferring a distinctive sweet-umami character [12]. *C. sikamea* was additionally characterized by an exceptionally high taurine content (2046 mg 100 g^−1^); while tasteless per se, taurine modulates cellular osmotic pressure and cholesterol metabolism, and may indirectly influence flavor stability during post-harvest storage [24]. Arginine (Arg) imparted a subtle bitterness (mean TAV = 8.05); however, previous studies indicate that such low-level bitterness is effectively masked by co-existing AMP and monosodium glutamate, resulting in a balanced and enduring savory impression [8]. Glycine (Gly) played a comparable modulatory role (mean TAV = 2.81) and is known to potentiate sweetness perception via allosteric modulation of the T1R2/T1R3 sweet receptor.

The variation in FAA signatures is likely attributable to the specific trophic and environmental regimes—including feeding strategy, salinity, and temperature—experienced by each bivalve species [36,37]. Seasonal profiling of *R. philippinarum* and *M. meretrix* has further demonstrated that Glu, Asp, Gly and Ala levels peak during periods of phytoplankton blooms. This finding corroborates our results and strongly underscores the trophic dependency of these umami metabolites [36]. Collectively, our data reinforce the consensus that Glu and Asp are the principal chemical drivers of bivalve palatability, while Ala, Gly and Arg serve as auxiliary modulators that shape the unique flavor architectures of each species.

### 4.2. Comparative Analysis of Nucleotide Composition of Six Bivalves

In the present study, 5′-guanosine monophosphate (GMP) was identified as the predominant nucleotide contributing to umami taste, with taste activity values (TAVs) ranging from 15.72 to 21.21 across the six bivalve species. A notable synergistic effect was observed between GMP and glutamate (Glu), which significantly enhanced umami intensity, consistent with previously reported mechanisms [9]. This synergy aligns with the Yamaguchi model [15], which posits that a 1:1 ratio of GMP to monosodium glutamate (MSG) can amplify umami intensity by approximately 30-fold. This mechanistic insight elucidates the characteristically intense umami flavor of *C. sikamea*, regionally known as “milk in the sea.” Among the species studied, *M. meretrix* exhibited the second-highest equivalent umami concentration (EUC), corroborating its culinary reputation as a delicacy frequently used in soups and dried powder formulations [12]. Inosine monophosphate (IMP) was not detected in any samples, likely due to rapid enzymatic degradation by phosphatases post-sampling [38], a phenomenon also observed in heat-treated bivalves [3]. The presence of IMP in bivalves remains a subject of debate; while some studies report only GMP in oysters, others have detected IMP in frozen clam species, suggesting a potential degradation pathway from ATP to IMP via AMP [39,40,41]. Although AMP was detected at low levels (TAV > 1) in the clam species studied and is known to exert a subsidiary umami-enhancing effect [5,6], its content may be subject to seasonal fluctuations linked to water temperature-regulated metabolic activity [27]. Ultimately, the distinct profiles of free amino acids and nucleotides across bivalve species constitute a complex flavor system that underpins their unique sensory characteristics [42,43,44].

### 4.3. Potential Regulation Mechanism of Six Bivalvesumami Genes

Based on the observed interspecific variations in umami compound profiles, we hypothesize that differences in synthesis arise from the divergent expression of core metabolic genes and the functional evolution of their key protein domains. Supporting this hypothesis, a previous study on four geographic populations of *M. meretrix* in Jiangsu Province reported stable inheritance of umami-related traits—such as glutamate and AMP content—across parental and offspring generations, suggesting a strong genetic basis for umami formation [45,46,47,48]. qRT-PCR analysis revealed that the expression level of the glutamate dehydrogenase gene (*GLUD1*) in *C. sikamea* was 3.2-fold higher than in *S. constricta*. This differential expression may be attributed to the enhanced binding affinity of *GLUD1* to NAD(P)^+^ cofactors via allosteric residues Glu152 and Arg147, potentially facilitating glutamate accumulation [49]. Conversely, mutations at Arg147, Glu152, and Ala161 in *S. constricta* may impair glutamate synthesis. Similarly, expression of *HPRT1* was 6.5-fold higher in *S. constricta* and *R. philippinarum* compared to *C. sikamea*, possibly due to structural alterations resulting from mutations at Glu159 and Glu169 [18]. Regarding *ADSS*, the disulfide bond involving Cys98 is critical for thermal stability; its disruption may reduce enzymatic efficiency, indirectly influencing glutamate synthesis [50]. Furthermore, a mutation at Lys372 within the conserved pyridoxal phosphate (PLP)-binding motif “KLKKY” of *GOT1* suggests a potential shift in substrate specificity. This alteration may link the enzyme to aspartate-mediated regulation of glutamate metabolism, reflecting functional divergence among bivalve species [17,33].

In summary, this study identifies four candidate genes—*GLUD1*, *HPRT1*, *ADSS*, and *GOT1*—as potential regulators of umami compound synthesis in six economically important bivalve species. Elucidating their molecular mechanisms may provide actionable targets for flavor-oriented genetic breeding in shellfish [10]. However, several limitations should be noted. First, the Equivalent Umami Concentration (EUC) serves primarily as a chemical index for potential umami intensity. Although sample quality was optimized, actual sensory perception involves complex interactions and is constrained by psychophysical factors such as taste saturation. Consequently, higher EUC values indicate greater umami potential but may not translate linearly to perceived intensity. Second, regarding the correlation analysis between metabolites and gene expression, unadjusted *p*-values were utilized to minimize Type II errors in this exploratory context. To ensure robustness, emphasis was placed on associations with high correlation coefficients (|r| > 0.8) and biological relevance. However, several limitations should be noted. First, the Equivalent Umami Concentration (EUC) serves primarily as a chemical index for potential umami intensity. Although sample quality was optimized, actual sensory perception involves complex interactions and is constrained by psychophysical factors such as taste saturation. Consequently, higher EUC values indicate greater umami potential but may not translate linearly to perceived intensity. Second, regarding the correlation analysis between metabolites and gene expression, unadjusted *p*-values were utilized to minimize Type II errors in this exploratory context. To ensure robustness, emphasis was placed on associations with high correlation coefficients (|r| > 0.8) and biological relevance.

## 5. Conclusions

In this study, the taste characteristics of six economically important shellfish species and their underlying molecular regulatory mechanisms were evaluated. The results demonstrated that glutamic acid is the primary contributor to umami intensity, exhibiting a significant synergistic effect with GMP. Furthermore, gene expression analysis identified *GLUD1* as a key potential regulator governing the metabolism of umami substances. Collectively, these findings provide a scientific basis for the objective evaluation of shellfish flavor quality and establish a theoretical foundation for the development of premium seafood flavorings and genetic breeding strategies.

## Figures and Tables

**Table 1:** Sampling period and umami peak period of six bivalves.

Species	Peak Peridos	Reference Basis
*Ruditapes philippinarum*	November	Glycine ↑ [20]
*Meretrix meretrix*	May	Glutamate ↑ [21]
*Cyclina sinensis*	September	Glutamate ↑ [22]
*Mercenaria mercenaria*	October	Glycine ↑ [23]
*Crassostrea sikamea*	February	Succinic acid ↑ [24]
*Sinonovacula constricta*	April	Glutamate ↑ [25]

**Table 2:** Primer information of umami-related genes.

Gene ID	Gene Name	Primer Information
XM-060710013.1	*RpGOT1*	F: CGAACGTGTGGGTAACTTGTG R: CCCGTTCAATGCTGGGTTGT
Hic-asm_15.687	*CsGOT1*	F: GGCAAGCCATGGGTTTTACC R: CAGCCAACCTGATAGCACCA
evm.TU.Chr1.1678	*MmGOT1*	F: AAGCCTTGGGTGTTGCCT R: AATCTGCCGCTAACCTGAGT
evm.TU.ptg000007l.378	*CpGOT1*	F: AAGGCAGCACCAGAAAAGAC R: TCCAAGTTACCAGAAGCGAA
Sco05g09590.1	*ScGOT1*	F: GTGAAGGCTCGTGTGATGTCTC R: GCAGCGTTGTTTAGGATGGTG
XM-045332602.2	*MeGOT1*	F: TGACTGCGCTTACCAAGGAT R: CACAGGTTTCCAACACGCTC
XM-060702916.1	*RpGLUD1*	F: ACCGTGTCATATTTCGAGTGGT R: CAGCAATACGCTCAGCAAAGT
Hic-asm12.361	*CsGLUD1*	F: ACTACCGACCAATCAGCAGC R: TCCTCCTCCAGCCCAAAT
evm.TU.Chr14.701	*MmGLUD1*	F: AGGGTTTTGGTAATGTGGGT R: CAAGTTGCCTTCATACGGTT
evm.TU.ptg000008l.359	*CpGLUD1*	F: GCCTTGGCTGCCCTAAT R: CTCATCTCCCTCTCTCGTGT
Sco17g03900.1	*ScGLUD1*	F: GAATGAAGATGAGGTGAACGC R: TACCCATGTCTGGAGCAGG
XM-045356148.2	*MeGLUD1*	F: GAATTTTGCTGCAAGCATCGC R: AAAGGTCAGACCAGCGTTGT
XM-060699380.1	*RpADSS*	F: CAGGTGGATTCCCAACAGA R: GTTTAGTGATTGCCAACGCT
Hic-asm-10.1590	*CsADSS*	F: AAGACGTGTAGGCTGGTTGG R: TCGCTGGAAAACTGTCCTCG
evm.TU.Chr13.1848	*MmADSS*	F: CCGACGTCGTGTGTAGATGT R: TCCAACTTCTTCGGCAGCTT
evm.TU.ptg000008l.380	*CpADSS*	F: TCCCCATAAAAAGCCTAGAACA R: CCCTGACAGCGACAGCAAA
Sco14g15190.1	*ScADSS*	F: TCACGAGCAACACAACGGC R: CCCAACACGACAGAAACGC
Gene-LOC123545001	*MeADSS*	F: TATGGTGGAGGGAGCACAGT R: CACCTGACCCAACCCTTGTT
XM-060697710.1	*RpHPRT1*	F: AGGGATTAGTGGCTCTGTGTG R: ACCTGACTGCTTGTCATCCAC
Hic-asm-3.404	*CsHPRT1*	F: AACCTTGAGAACCTGGCTGG R: TTTGTAGCCGACACTTCGCT
evm.TU. Chr8.253	*MmHPRT1*	F: CAACGTCGGGCTATATTGTGC R: AGCACACACAGCGCTACTAA
evm.TU.ptg000009l.10	*CpHPRT1*	F: TCCCACCCCCACAAAAG R: GGTCACGAGGACAGCAAAG
Sco04g04350.1	*ScHPRT1*	F: GGTCGCCCTATGTGTCTTG R: TCTCCCCTTCTGGCTTGTC
XM-045312230.2	*MeHPRT1*	F: TGAGACCTCAACACCACTTGC R: GGTGCGGCCAGTGTCTATAAT
Rp-reference gene	*RpL3*	F: ACCATCAACCTTCACAAGAGG R: TGTTTCTACTCGGACATCTGG
Cs-reference gene	*β-actin-1*	F: CACCACAACTGCCGAGAG R: CCGATAGTGATGACCTGACC
Mm-reference gene	*β-actin-2*	F: TTGTCTGGTGGTTCAACTAT R: TCCACATCTGCTGGAAGGTG
Cs-reference gene	*EF1α-1*	F: AGTCACCAAGGCTGCACAGAAAG R: TCCGACGTATTTCTTTGCGATGT
Sc-reference gene	*RS9*	F: TGAAGTCTGGCGTGTCAAGT R: CGTCTCAAAAGGGCATTACC
Me-reference gene	*EF1α-2*	F: AGTCGGTCGAGTTGAAACTGGTGT R: TCAGGAAGAGACTCGTGGTGCATT

Rp: *R. philippinarum*, Cs: *C. sinensis*, Mm: *M. meretrix*, *Cp*: *C. sikamea*, Sc: *S. constricta*, Me: *M. mercenaria*.

**Table 3:** Total free amino acid (TFAA) content in six bivalve species.

Amino Acid	Content (mg/100 g)					
	*M. meretrix*	*C. sinensis*	*M. mercenaria*	*R. philippinarum*	*C. sikamea*	*S. constricta*
Total free amino acid	3932.45 ± 230.30 ^ab^	4400.31 ± 235.00 ^bc^	4815.79 ± 1036.34 ^c^	4217.96 ± 637.97 ^ab^	4851.66 ± 852.28 ^c^	3783.93 ± 139.18 ^a^

Different letters (a–c) within the same row indicate statistically significant differences based on Tukey’s HSD test (*p* < 0.05). Values sharing the same letter are not statistically different.

**Table 4:** Contents of flavor nucleotides in six bivalve species.

Nucleotide	Content (mg/100 g)					
	*M* *. meretrix*	*C* *. sinensis*	*M* *. mercenaria*	*R* *. philippinarum*	*C* *. sikamea*	*S* *. constricta*
GMP	253.83 ± 27.46 ^a^	196.48 ± 19.44 ^c^	243.11 ± 25.96 ^ab^	203.02 ± 9.93 ^bc^	265.17 ± 100.1 ^a^	226.08 ± 22.66 ^abc^
AMP	111.14 ± 46.93 ^a^	51.08 ± 9.08 ^b^	131.29 ± 21.75 ^ac^	64.26 ± 14.1 ^b^	152.49 ± 66.28 ^c^	31.34 ± 17.04 ^b^

Different letters (a–c) within the same row indicate statistically significant differences based on Tukey’s HSD test (*p* < 0.05). Values sharing the same letter are not statistically different.

**Table 5:** TAVs of six bivalves flavor nucleotides.

Nucleotide	*M* *. meretrix*	*C* *. sinensis*	*M* *. mercenaria*	*R* *. philippinarum*	*C* *. sikamea*	*S* *. constricta*
GMP	20.31 ± 2.20 ^a^	15.72 ± 1.56 ^c^	19.45 ± 2.08 ^ab^	16.24 ± 0.79 ^bc^	21.21 ± 8.01 ^a^	18.09 ± 1.81 ^abc^
AMP	2.22 ± 0.94 ^a^	1.02 ± 0.18 ^b^	2.63 ± 0.44 ^ac^	1.29 ± 0.28 ^b^	3.05 ± 1.33 ^c^	0.63 ± 0.34 ^b^

TAV represents the ratio of concentration to taste threshold. Values are expressed as means ± SD (*n* ≥ 3). Different superscript letters within the same row indicate significant differences (*p* < 0.05, Tukey’s HSD).

**Table 6:** Equivalent Umami Concentrations (EUC) of six bivalve species.

Species	EUC (g MSG/100 g Dry Weight)
*M*. *meretrix*	329.56 ± 35.60 ^ab^
*C*. *sinensis*	283.23 ± 28.05 ^bc^
*M*. *mercenaria*	284.93 ± 30.20 ^bc^
*R*. *philippinarum*	174.32 ± 8.54 ^d^
*C*. *sikamea*	449.35 ± 169.41 ^a^
*S. constricta*	214.08 ± 21.40 ^cd^

EUC represents the Equivalent Umami Concentration (g MSG/100 g dry weight). Data are presented as mean ± SD (*n* ≥ 3). Different superscript letters within the column indicate statistically significant differences (*p* < 0.05) based on Tukey’s HSD test.

**Figure A1:**
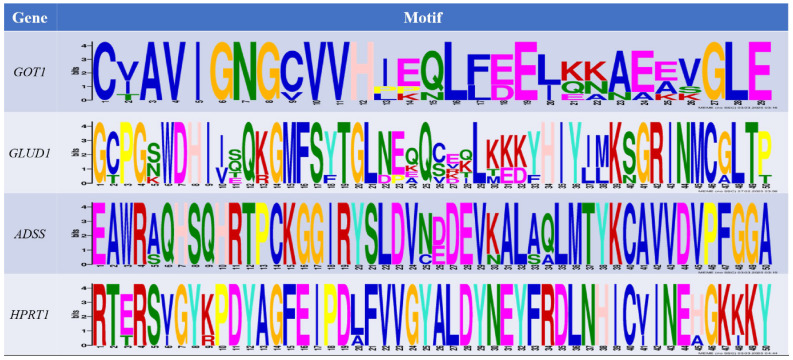
Conserved functional motifs of four metabolism-essential genes (*GOT1*, *GLUD1*, *ADSS* and *HPRT1*).

## Data Availability

The original contributions presented in this study are included in the article. Further inquiries can be directed to the corresponding authors.

## Appendix A

**Table A1:** Sample details and key biological traits of the six bivalve species.

Species	Live Weight/g	Soft Weight/g	Shell Length/mm	Shell Meat Ratio
*M. meretrix*	24.38 ± 2.97	10.30 ± 1.42	43.83 ± 2.15	1.37 ± 0.12
*C. sinensis*	13.59 ± 1.09	7.36 ± 0.75	33.13 ± 1.16	0.85 ± 0.11
*M. mercenaria*	26.91 ± 2.00	11.00 ± 1.01	44.00 ± 1.05	1.45 ± 0.15
*R. philippinarum*	10.21 ± 2.68	5.26 ± 1.47	37.02 ± 2.85	0.97 ± 0.28
*C. sikamea*	14.90 ± 4.13	3.91 ± 0.78	44.42 ± 5.47	2.87 ± 1.10
*S. constricta*	12.27 ± 1.02	9.22 ± 0.75	55.93 ± 1.91	0.33 ± 0.037
